# Alginate-immobilized exopolymeric substances from bacteria for Lead (Pb) removal: biosorption and reusability studies

**DOI:** 10.1007/s10532-026-10280-3

**Published:** 2026-03-17

**Authors:** Adeline Su Yien Ting, Kai Hao Tiew, Keang Peng Song

**Affiliations:** School of Science, Monash University Malaysia, Jalan Lagoon Selatan, 47500 Bandar Sunway, Selangor Darul Ehsan Malaysia

**Keywords:** Column biosorption, EPS, Fixed-bed column, Heavy metals, Regeneration, Wastewater

## Abstract

In this study, exopolymeric substances (EPS) from *Bacillus cereus* were immobilized in alginate to form alginate-EPS beads. The surface characteristics of the alginate-EPS beads were examined using Scanning Electron Microscopy (SEM) and Fourier-Transform Infrared Spectroscopy (FTIR). The mechanisms of Pb removal was determined based on equilibrium and kinetic models. Packed-bed biosorption studies using Pb solutions (750 mg L^−1^ concentration) evaluated their efficacy for Pb uptake and removal, under the influence of initial pH of metal solution and the size of biosorbents. The reusability of the alginate-EPS beads was also tested by examining the column regeneration, exhaustion time and sorption–desorption activities of the Pb-loaded beads. Results revealed that the alginate-EPS beads produced have uneven surface with various functional groups (hydroxyl, amide, carboxyl, phosphates) detected. Their Pb removal efficacy was highest in Pb solutions with pH 4, recording 51.52% Pb removal, followed by pH 6 (48.62%) and pH 8 (46.78%). For bead size, alginate-EPS beads measuring 3 mm diameter size was more efficient in Pb removal (51.52%), compared to 2 mm- (39.23%) and 5 mm-sized beads (28.52%). All alginate-EPS beads complied with the Langmuir isotherm (R^2^ = 0.992) and pseudo-second order kinetic (R^2^ = 0.995), suggesting monolayer adsorption is likely to have occurred on their homogenous surface. The alginate-EPS beads were successfully used for five adsorption cycles, with consistent Pb removal (41.40–50.59%). This study recommends the use of 3 mm bead size for Pb removal (pH 4 solutions) due to greater Pb removal (51.5%) and uptake (13.29 mg g^−1^), and reasonable exhaustion time (540 min). There is potential of applying alginate-EPS beads in packed-bed systems as a feasible and effective method for the removal of heavy metals from wastewater.

## Introduction

Heavy metals are a threat to the environment as they are found in untreated industrial wastewaters. One such example of toxic heavy metal is Lead (Pb). Lead (Pb) is a heavy metal that is commonly found in the industrial wastewaters associated with the manufacturing of paints and textiles (Johnson et al. 2009). Pb pollution in the environment can lead to harmful effects impacting aquatic life, terrestrial plants, and eventually humans when ingested. Pb poisoning has been reported and is linked as possible causes of anaemia, brain damage, and kidney damage (Ting et al. 2025). Conventional technologies to remove Pb and other heavy metals from wastewater include ion exchange and chemical precipitation, but they are costly and generate secondary waste (Fu and Wang 2011). As an alternative, bio-based materials are explored as a more economical and environmentally-friendly approach to remove heavy metals from wastewater.

Bio-based materials can include microbial biomass and their by-products (Bishnoi and Garima 2005; Nguyen et al. 2013). Various microbial species have been studied for their ability to remove heavy metals from aqueous phase using the biosorption approach. Biosorption is a physicochemical process whereby heavy metals from the solution are retained through the binding of the heavy metal ions to the functional groups present on the surface of the bio-based materials (biosorbent) (Kim et al. 2006; Liu et al. 2021). This metal binding on biosorbents occurs through the electrostatic attraction between positively charged metal ions and negatively charged functional groups on the biosorbent (Ding et al. 2019).

In recent years, microbial exopolymeric substances (EPS) have become increasingly popular for heavy metal removal. EPS are biopolymers produced by microbes mainly for biofilm formation and protection against environmental stresses (Flemming 2011). EPS harbour many organic functional groups that are able to adsorb and chelate heavy metal ions, which makes them suitable as biosorbents for the removal of heavy metals (Koechler et al. 2015). Several biosorption studies have revealed that microbial EPS have the potential of removing heavy metals with up to 90% removal efficacy (Mubashar and Faisal 2012; Shameer 2016; Cheah et al. 2022a). To enhance metal biosorption by EPS, efforts to embed EPS in alginate beads have been employed in recent studies (Ozdemir et al. 2005; Kumari et al. 2017; Ajao et al. 2020). This EPS-embedding process encapsulates the EPS within alginate beads to form alginate-EPS beads, which improves the mechanical strength and stability of EPS (Li et al. 2022). A study demonstrated that the removal of Cu, Ni, Cd, and Co was increased by 8, 2, 9, and 6%, respectively, with the use of alginate-EPS beads, compared to using pure alginate beads (Ozdemir et al. 2005). Similarly, alginate-EPS beads were revealed to have Pb removal efficacy of 416.67 mg g^−1^, which was 3.5 folds higher than the efficacy of pure alginate beads (Kumari et al. 2017).

The biosorption mechanism of the alginate-EPS beads is best studied using adsorption isotherm and kinetic models. These models are typically used to study adsorbent-adsorbate relationship that determines the maximum adsorption capacity and optimum equilibrium time (Kumar et al. 2016). Applying these models on the alginate-EPS beads will elucidate their biosorption mechanism for heavy metal removal, which helps to optimize the use of these beads. Most biosorption studies using alginate-EPS beads are performed in batch systems. By contrast, application of the beads in a packed-bed system is rarely reported although packed-bed columns are more practical and manageable. This is because a packed-bed biosorption system is capable of treating larger volumes of wastewater through continuous flow, and is more applicable and useful in treating industrial wastewaters (Papirio et al. 2017). It is therefore pertinent that important parameters that influence the removal of heavy metals in packed-bed systems are optimized to maximize the potential of using alginate-EPS beads for biosorption.

One of the parameters that heavily influences biosorption in a fix-bed system is the pH of the metal solution (influent) (Kumar et al. 2016). Heavy metals are known to remain in their ionic state under acidic conditions and precipitate in alkaline conditions (Naja and Volesky 2011), therefore, the pH of the influent metal solution is critical to achieve the maximum removal of heavy metals in a packed-bed system. Another important but often neglected parameter, is the size of biosorbents used in a packed-bed system. The size of biosorbents influence the packing density within the packed-bed system, consequently influencing the flow of metal solution and the interaction time of metal ions with the biosorbents (Hatzikioseyian et al. 2001). A highly-desirable packed-bed system should also have high reusability potential derived from simple sorption–desorption-sorption cycles. A metal-saturated packed-bed column can be regenerated by desorption of the adsorbed metals using acidic agents such as hydrochloric acid, nitric acid, and sulfuric acid (Rangsayatorn et al. 2004; Vijayaraghavan et al. 2005; Khitous et al. 2016). After regeneration, the packed-bed column can then be reused for subsequent cycles for metal removal. This keeps the packed-bed system sustainable as the same biosorbents can be reused multiple times prior to disposal.

In this study, EPS were collected and extracted from *Bacillus cereus* cultures and integrated in alginate beads to form alginate-EPS beads for heavy metal removal studies. The morphology and functional groups of the alginate-EPS beads were characterized through Scanning Electron Microscopy (SEM) and Fourier-Transform Infrared Spectroscopy (FTIR). Isotherm and kinetic studies were then carried out to determine the mechanisms of biosorption. Biosorption of Pb in a packed-bed system was performed using the alginate-EPS beads as biosorbents. Effects of important parameters including pH of influent metal solution and the size of the alginate-EPS beads, as well as the reusability of the packed-bed column, were evaluated. Our study therefore, was aimed at achieving several new findings. Firstly, to establish the potential of using alginate-EPS beads for heavy metal removal by elucidating the novel role of EPS in enhancing the functionality of alginate beads in metal biosorption. Secondly, there is also novelty in investigating the metal removal efficacy of the EPS-alginate beads in a packed-bed column system, a relatively new approach where EPS is used to enhance alginate beads that were later packed into bed columns for Pb removal. And thirdly, there are also significant new discoveries in determining the reusability (column regeneration) of the alginate-EPS beads packed-bed column for Pb removal. The findings reported in this study will bridge the current gap in using packed-bed biosorption systems, as well as presenting the new alginate-EPS beads in a packed-bed column system as a possible alternative for wastewater treatment in future.

## Methodology

### Production and extraction of EPS

The bacterial isolate *Bacillus cereus* was selected for the production of EPS as the EPS from this isolate recorded the highest Pb removal compared to EPS from several other bacterial species (Cheah et al. 2022a). Cultures of *B. cereus* was cultivated in conical flasks containing nutrient broth (25 °C, 120 rpm, 48 h) and then centrifuged (7000 rpm, 20 min) to separate the cells from the soluble EPS. The supernatant was then added to three parts of cold ethanol (95%) and left overnight at 4 °C to precipitate the EPS (Ziadi et al. 2018). The mixture was then centrifuged (7000 rpm, 30 min), the supernatant discarded, while EPS pellet was redissolved in 5 mL ultrapure water. The dissolved EPS was lyophilized (− 80 °C) to collect the crude EPS powder (Chug et al. 2016).

### Formation and characterization of hybrid alginate-EPS beads

A solution mixture of 1.5% w/v sodium alginate and 1.0% w/v crude EPS was prepared and added in a drop-wise manner into 3% w/v CaCl_2_ solution to form the alginate-EPS beads (Ozdemir et al. 2005). The beads were left to cure in the CaCl_2_ solution for 1 h, followed by rinsing thoroughly with distilled water. Alginate-EPS beads were freshly prepared for each of the assessments in the study. Prior to this, a separate set of alginate beads immobilized in various concentrations of EPS (0.5, 1.0, 1.5, and 2.0%) was performed by repeating the procedure. Plain alginate beads (0.0% EPS) were also prepared by substituting with 1.0% v/v sterile distilled water.

The freshly prepared alginate-EPS beads were characterized to evaluate the surface morphology and to identify the functional groups. The surface morphology of the alginate-EPS beads was analysed using Scanning Electron Microscopy (SEM). To prepare for the analysis, the beads were coated with gold using a sputter coater (Quorum Technologies Q150RS) with a sputter current of 30 mA for 40 s (Memon et al. 2008). The surface morphology of the coated specimens was then viewed using a Field-Emission Scanning Electron Microscope (FE-SEM) (Hitachi SU8010) with an accelerating voltage of 15 kV and a magnification of 30–1000 × (Memon et al. 2008).

The functional groups present on the alginate-EPS beads were identified using Fourier-Transform Infrared Spectroscopy (FTIR) (Perkim Elmer). To generate the FTIR spectrum, the freshly prepared alginate-EPS beads were scanned (16 scans) with resolution of 4 cm^−1^ and over a spectral range of 4000–400 cm^−1^ (Chew and Ting 2016). The functional groups on the alginate-EPS beads were then identified by assigning peaks detected in the spectra to functional groups according to referenced literatures (Maquelin et al. 2002; Liang et al. 2010; Vimalnath and Subramanian 2018).

### Biosorption of Pb ions by alginate-EPS beads

Biosorption experiments were conducted using alginate-immobilized EPS beads comprising of various EPS concentrations (0.5, 1.0, 1.5, and 2.0% w/v) and plain alginate beads (0% EPS) prepared. Pb metal solution was first prepared by dissolving Pb(NO_3_)_2_ salts (Friendemann Schmidt) in distilled water to derive Pb solution with a concentration of 50 mg L^−1^. The alginate-immobilized EPS beads and plain alginate beads (0.5 g fresh weight) were added into separate 50 mL Falcon tubes containing 30 mL of Pb solution (50 mg L^−1^) to initiate the biosorption experiment (agitated at 120 rpm, 24 h, 25 °C) (Cheah et al. 2022c). The residual amount of Pb remaining in the solution after 24 h incubation was determined using the Atomic Absorption Spectrophotometer (AAS) (Perkin Elmer Analyst 100) at a wavelength of 283 nm. The percentage of Pb removed from solution is calculated using Eq. 1.

1 $$\text{Pb removal percent} \left(\%\right)= \frac{\text{Initial Pb concentration}- \text{Final Pb concentration}}{\text{Initial Pb concentration}} \times 100\%$$

### Biosorption mechanisms

The biosorption mechanisms of the alginate-EPS beads were determined using isotherm and kinetic modelling studies. The isotherm models used in this study were the Freundlich and Langmuir models (Tyagi et al. 2020). Correlation coefficients (R^2^) from both models were compared to assess the biosorption mechanism of alginate-EPS beads. A higher R^2^ value for the corresponding isotherm indicates the biosorption process exists as either a monolayer adsorption (following the Langmuir isotherm) or multilayer adsorption (following the Freundlich isotherm). To establish the isotherm models, Pb stock solution (500 mg L^−1^) was first prepared by dissolving 0.8 g of Pb(NO_3_)_2_ salt (Friendemann Schmidt) in 1 L of distilled water. The biosorption experiments were initiated by adding 0.5 g fresh weight of alginate-EPS beads into 50 mL Falcon tubes containing 30 mL of Pb solution with concentrations varied from 50 to 500 mg L^−1^ (diluted accordingly from stock solution). The solutions were incubated (120 rpm, 25 °C, 6 h) and the residual Pb in the solutions were determined using the Atomic Absorption Spectrophotometer (AAS) (Perkin Elmer Analyst 100) at a wavelength of 283 nm (Cheah et al. 2022a). The experimental data collected was used to model the Freundlich (Eq. 2) and Langmuir (Eq. 3) isotherm models (Kumar et al. 2016), where q_e_ is the amount of Pb adsorbed at equilibrium (mg g^−1^), C_e_ is the remaining Pb concentration at equilibrium (mg L^−1^), K_F_ is a constant related to the amount of metal adsorbed (L g^−1^), n is a constant that measures the sorption intensity, q_max_ is the maximum sorption capacity (mg g^−1^), and K_L_ is a parameter related to sorption energy.

2 $${{q}_{e}=K}_{F}{C}_{e}^{n}$$

3 $${q}_{e}= {q}_{max}\frac{{K}_{L}{C}_{e}}{1+{K}_{L}{C}_{e}}$$

The kinetic models used in this study were the pseudo-first order and pseudo-second order kinetic models (Ablouh et al. 2019). Correlation coefficients (R^2^) from both models were compared to assess the biosorption mechanism of the alginate-EPS beads. A higher R^2^ value for the corresponding kinetic model indicates the biosorption process exists as either a physical attraction (following the pseudo-first order model) or a chemical attraction (following the pseudo-second order model). To test the kinetic model, biosorption experiments were conducted by adding 0.5 g fresh weight of alginate-EPS beads into 50 mL Falcon tubes containing 30 mL of Pb solution (50 mg L^−1^). The solutions were incubated (120 rpm, 25 °C, 6 h) and the kinetic data was obtained at designated time points (every 60 min) by measuring the concentration of Pb in the solution using the Atomic Absorption Spectrophotometer (AAS) (Perkin Elmer Analyst 100) at a wavelength of 283 nm (Cheah et al. 2022a). The experimental data was modelled with the pseudo-first (Eq. 4) and pseudo-second (Eq. 5) order kinetic models (Simonin 2016), where q_t_ is the amount of Pb adsorbed at a given time point (mg g^−1^), q_e_ is the amount of Pb adsorbed at equilibrium (mg g^−1^), t is time (min), k_1_ represents the pseudo-first order rate constant (min^−1^), and k_2_ represents the pseudo-second order rate constant (g mg^−1^ min).

4 $${q}_{t}={q}_{e}\left[1-exp\left(-{k}_{1}t\right)\right]$$

5 $${q}_{t} = {q}_{e}^{2}\frac{{k}_{2}t}{1 + {k}_{2}{q}_{e}t}$$

### Packed-bed biosorption studies

#### Packed-bed biosorption experimental design

The detailed set-up for the packed-bed biosorption studies follows the description by Ting et al. (2025). Briefly, packed-bed biosorption studies were conducted in a glass column with an inner diameter of 2 cm. Glass beads were placed inside the column, beginning at the bottom of the column to a height of 1 cm to provide uniform flow and distribution of the metal solution (Hasan et al. 2009). A layer of glass wool (0.5 cm height) was placed on top of the glass beads to support the freshly-prepared alginate-EPS beads. The column was then filled with the alginate-EPS beads up to a 15 cm bed height. Another layer of glass wool (0.5 cm height) was placed on top of the alginate-EPS beads to secure the beads in place during biosorption.

#### Effect of initial pH of influent metal solution

To study the effect of initial pH of influent metal solution on biosorption, experiments were carried out by varying the pH of Pb solution to give rise to solutions with pH 4, 6, and 8 (Kumar et al. 2016). The metal solution was prepared by dissolving 2.4 g of Pb(NO_3_)_2_ salt (Friendemann Schmidt) in 2 L of distilled water to obtain Pb solution at a concentration of 750 mg L^−1^. The pH of the Pb solution was adjusted using either 1 M HCl or 1 M NaOH to the desired pH. To initiate the biosorption experiment, the Pb solution was fed to the packed column from the bottom, to flow in an upward direction using a peristaltic pump (Cole-Palmer Masterflex L/S 100RPM). The Pb solution was fed to the column at a flow rate of 3 mL min^−1^ for a total of 600 min. Effluent from the top of the column was collected periodically at every 60 min and measured for Pb levels using the AAS at a wavelength of 283 nm (Cheah et al. 2022a).

#### Effect of biosorbent size

To study the effect of biosorbent size on biosorption, experiments were carried out by varying the size of the alginate-EPS beads (2, 3, and 5 mm in diameter) (Kumar et al. 2016). The different sized beads were formed by extruding the alginate-EPS mixture into CaCl_2_ solution using syringes with tips of respective diameter sizes. The freshly prepared beads were then packed into the column (15 cm bed height) and the biosorption experiment was initiated as described in the earlier sections where Pb solution (750 mg L^−1^, pH 4) was fed to the column (3 mL min^−1^) for 600 min. Effluent from the top of the column was also collected every 60 min and analysed as described previously.

#### Column regeneration

Reusability of the packed-bed column was evaluated through column regeneration studies. Biosorption experiment was first performed as described previously using Pb solution (750 mg L^−1^, pH 4), and a column packed with 3-mm diameter alginate-EPS beads. After biosorption, desorption of the metal-saturated column was performed using acidified CaCl_2_ (1% w/v, pH 3) solution as the desorption agent (Volesky et al. 2003). To initiate the desorption process, the acidified CaCl_2_ solution was fed to the column (3 mL min^−1^) from the bottom, in an upwards direction for a total of 600 min. The effluent from the top of the column was collected at every 60 min and measured for Pb levels using the AAS. After desorption was completed, the column was reused for another biosorption cycle with the same conditions and procedures as described. A total of five adsorption cycles and four desorption cycles were carried out to test the reusability of the alginate-EPS beads in a packed-bed system.

The durability of the alginate-EPS beads throughout the sorption–desorption cycles were also assessed via SEM. SEM detects for changes to the surface of the alginate-EPS beads as a consequence of the continuous exposure to Pb solution and acidic desorption agent. Alginate-EPS beads were collected from stages prior to desorption, after the first desorption cycle, and after the fourth desorption cycle, for observations using SEM. The samples were coated with gold using a sputter coater (Quorum Technologies Q150RS) with a sputter current of 30 mA for 40 s. SEM was then performed to view their surface morphology using a field-emission scanning electron microscope (FE-SEM) (Hitachi SU8010) with an accelerating voltage of 15 kV and a magnification of 35–200 × (Memon et al. 2008).

#### Analysis of breakthrough curve

Results from the packed-bed biosorption studies were presented as breakthrough curves, where a normalized concentration ratio of the effluent (Pb concentration) to the initial Pb concentration (*C/C*_*o*_) is expressed versus time (*t*). The area under the breakthrough curve represents the total amount of metal adsorbed by the column (*q*_*total*_), which is calculated by integration using Eq. 6, where *Q* is the flow rate of the metal solution (mL min^−1^) and *C*_*R*_ is the concentration of metal removed (mg L^−1^) (Ronda et al. 2015). The area underneath breakthrough curves were analysed using GraphPad Prism version 9.

6 $${q}_{total}= \frac{Q}{1000} {\int }_{t=0}^{t={t}_{total}}{C}_{R} dt$$

To determine the percentage of Pb removed by the column, the total amount of Pb ions fed to the column (*m*_*total*_) was first calculated with Eq. 7 (Hasan et al. 2009), where *C*_*o*_ is the initial Pb concentration (mg L^−1^), and *t*_*ex*_ is defined as the time (min) when the Pb concentration in the effluent has reached 95% of the initial concentration (*C/C*_*o*_ = 0.95), otherwise known as column exhaustion time.

7 $${m}_{total}= \frac{{C}_{o}Q{t}_{ex}}{1000}$$

Then, the percentage of Pb removed by the column (Pb removal) was calculated as a ratio of total metal adsorbed (*m*_*ad*_) to total metal fed to column (*m*_*total*_) using Eq. 8 (Hasan et al. 2009).

8 $$Pb Removal= \frac{{m}_{ad}}{{m}_{total}} \times 100\%$$

The percentage of metal recovered from desorption (Pb recovery) was calculated as a ratio of total metal desorbed (*m*_*de*_) to total metal adsorbed (*m*_*ad*_) using Eq. 9 (Hasan et al. 2009).

9 $$Pb Recovery= \frac{{m}_{de}}{{m}_{ad}} \times 100\%$$

### Statistical analysis

Measurements for biosorption studies were performed using triplicates. Mean values are reported with standard deviations. The data obtained were analysed with One-Way Analysis of Variance (ANOVA) using the Statistical Package for the Social Sciences (SPSS) version 26.0. The mean values were analysed with Tukey’s Test (*p* < 0.01) to evaluate the statistical significance.

## Results

### Characterization of alginate-EPS beads

**Fig. 1:**
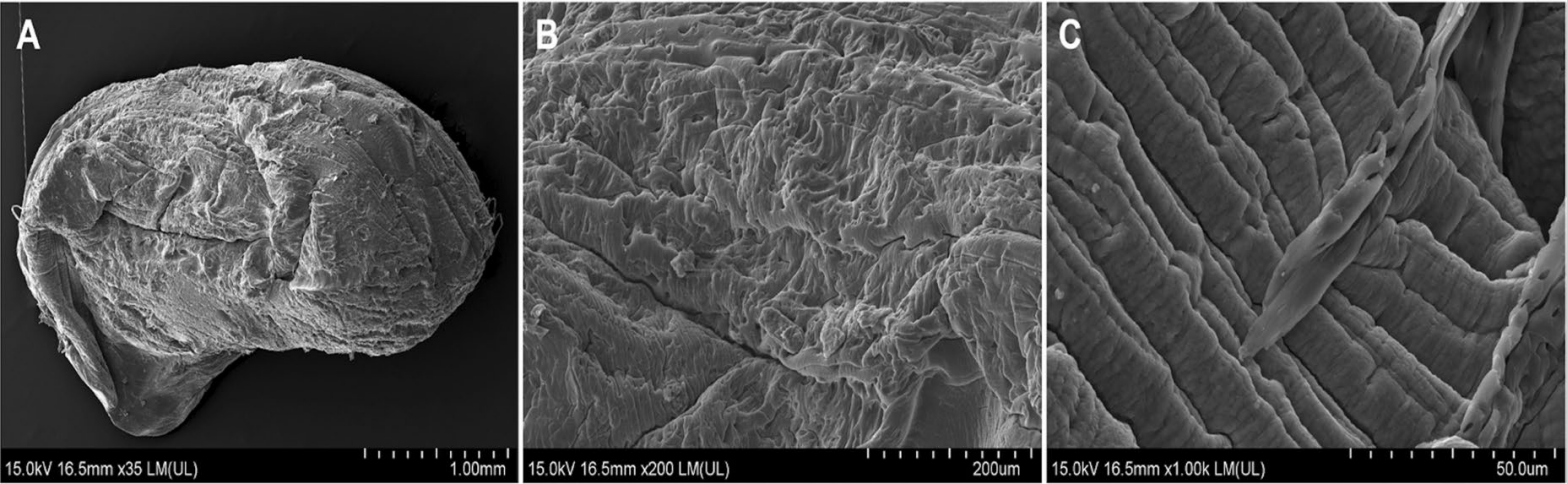
SEM micrographs of alginate-EPS bead at **A** 35 × magnification, **B** 200 × magnification, and **C** 1000 × magnification

**Fig. 2:**
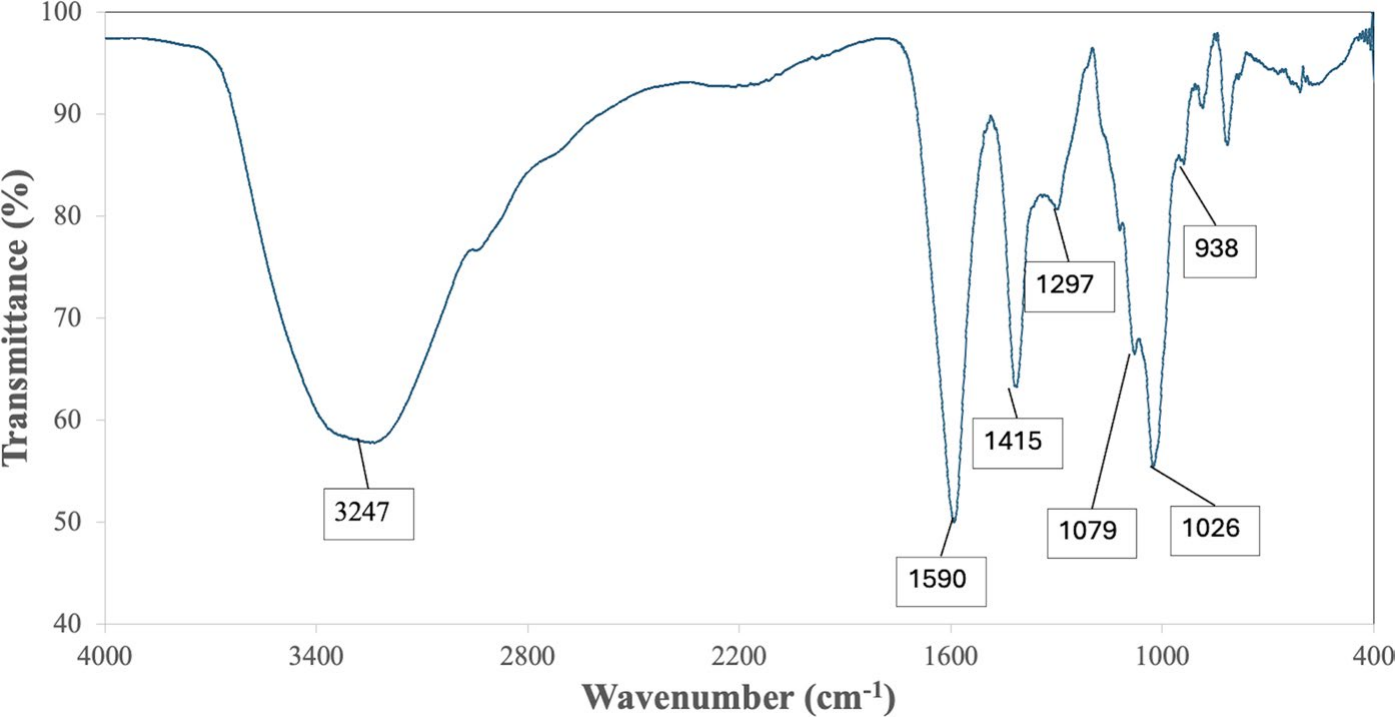
FTIR spectrum of alginate-EPS beads

Characterization studies of the alginate-EPS beads revealed interesting surface morphology and functional groups. The surface morphology of the alginate-EPS beads were rough in appearance (based on the SEM analysis) (Fig. 1A). At a magnification of 200 × , the surface of the beads appeared dense and highly uneven (Fig. 1B). At 1000 × magnification, uneven folds can be found on the surface of the beads, suggesting possible increased total surface area (Fig. 1C). The FTIR spectra revealed that the functional groups of the alginate-EPS include possible hydroxyl (–OH, at 3243 cm^−1^), amide (CONH_2_ at 1590 cm^−1^), carboxyl (COOH at 1415 cm^−1^), and phosphate (PO_4_^3−^ at 1297, 1079, 1026, and 938 cm^−1^) groups (Fig. 2). Overall, it can be deduced that the alginate-EPS beads have uneven surface morphology and harbour many organic functional groups.

### Biosorption of Pb ions by plain alginate and alginate-EPS beads

**Fig. 3:**
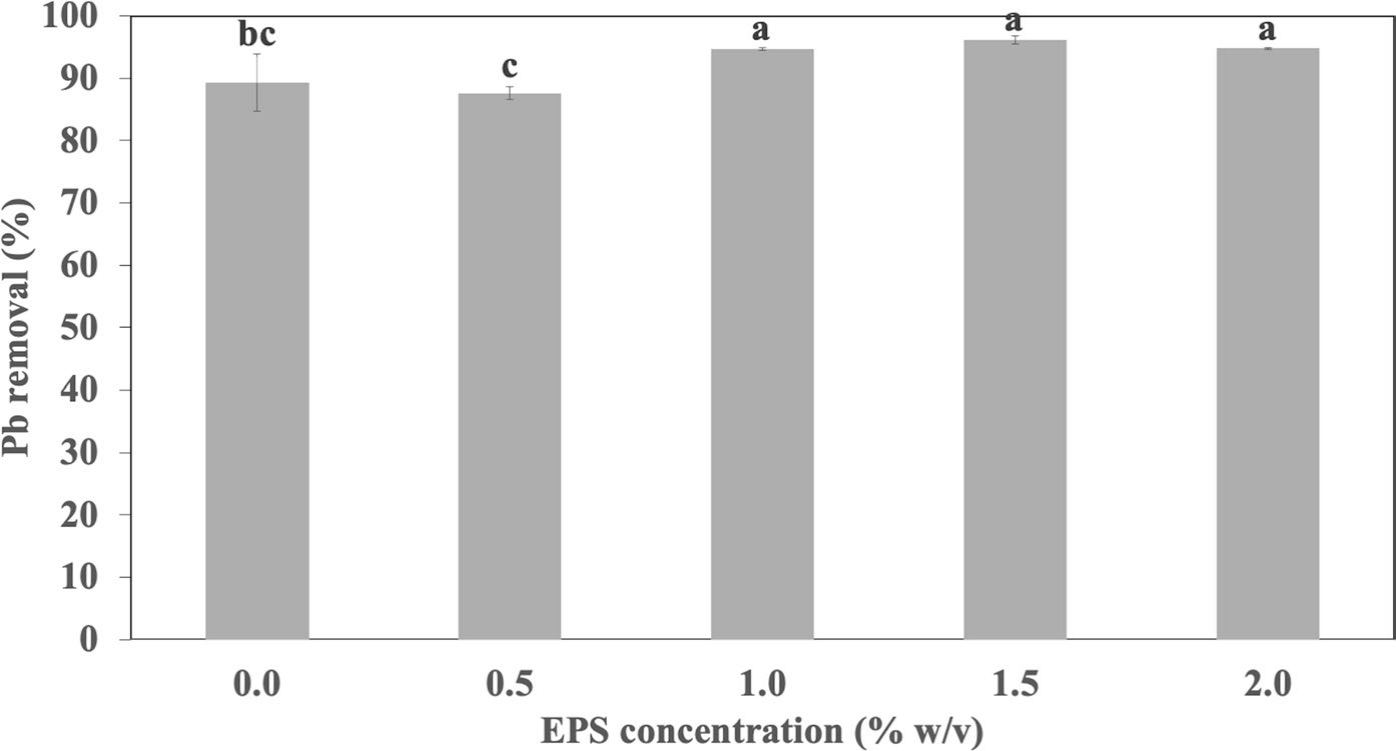
Lead (Pb) removal (%) by plain alginate beads (0% EPS) and alginate-EPS beads with varying concentrations of EPS (0.5, 1.0, 1.5, and 2.0% w/v EPS). Values are mean of triplicates. Means with the same letters indicate no significant difference according to Tukey comparisons (*p* < 0.01). Error bars indicate standard deviation of means

The biosorption of Pb by plain alginate beads and alginate-immobilized EPS showed that addition of 1.0% EPS achieved significantly higher removal of Pb (94.75%) compared to plain alginate beads (90.93%) and alginate beads with 0.5% EPS (87.60%) (Fig. 3). It is postulated that 0.5% EPS may be too little to render any influence to Pb biosorption, resulting in non-significant differences in amount of Pb removed compared to the plain alginate beads (control). When compared with alginate beads immobilized with 1.5 and 2.0% of EPS, no significant differences were detected from Pb removal recorded by alginate beads with 1.0, 1.5, and 2.0% EPS, with 94.75, 96.15, and 94.79% of Pb removal (Fig. 3). As such, subsequent biosorption studies were conducted using alginate-immobilized with 1.0% EPS concentration.

### Biosorption mechanisms

**Fig. 4:**
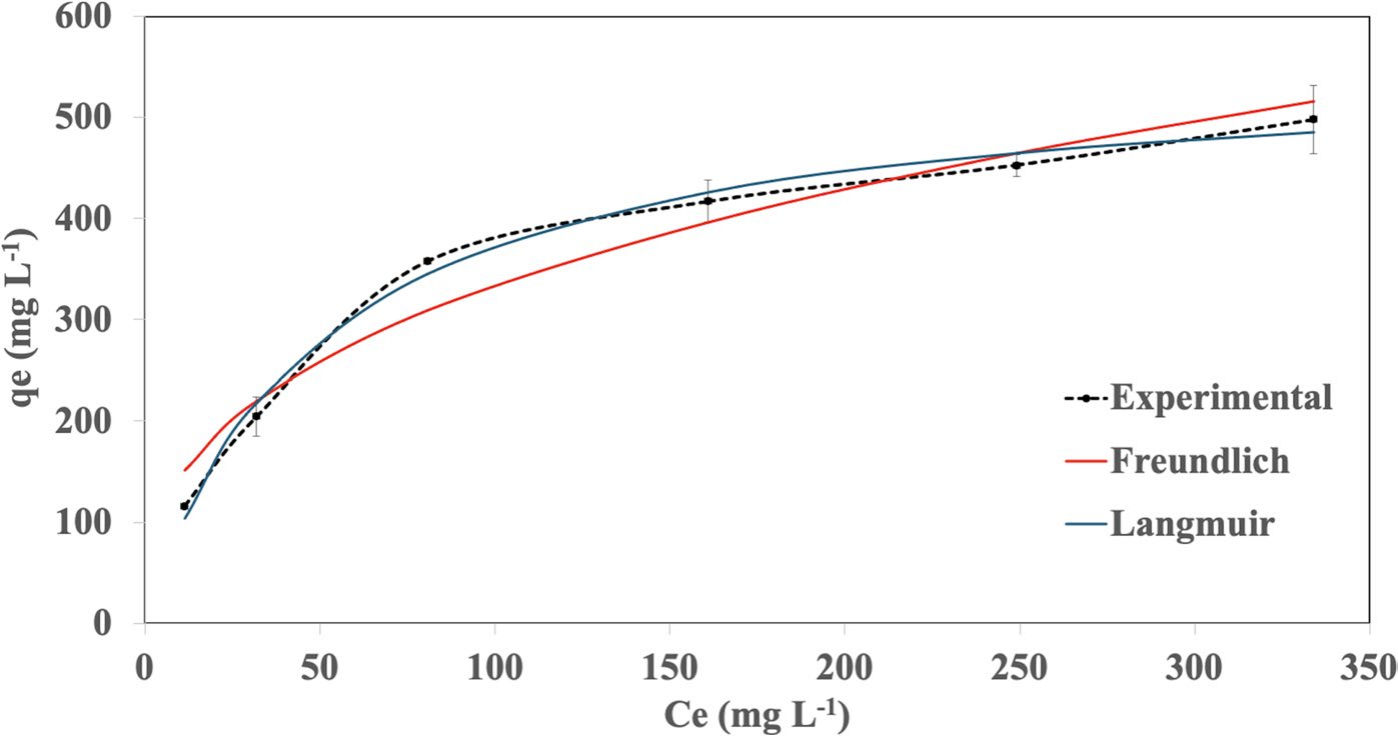
Biosorption isotherm models for alginate-EPS beads. Experimental data were fitted to the isotherm models through non-linear regression using the Microsoft Excel Solver function. Error bars indicate standard deviation of means. C_e_ represents the residual Pb concentration at equilibrium, and q_e_ represents the amount of Pb adsorbed at equilibrium

**Fig. 5:**
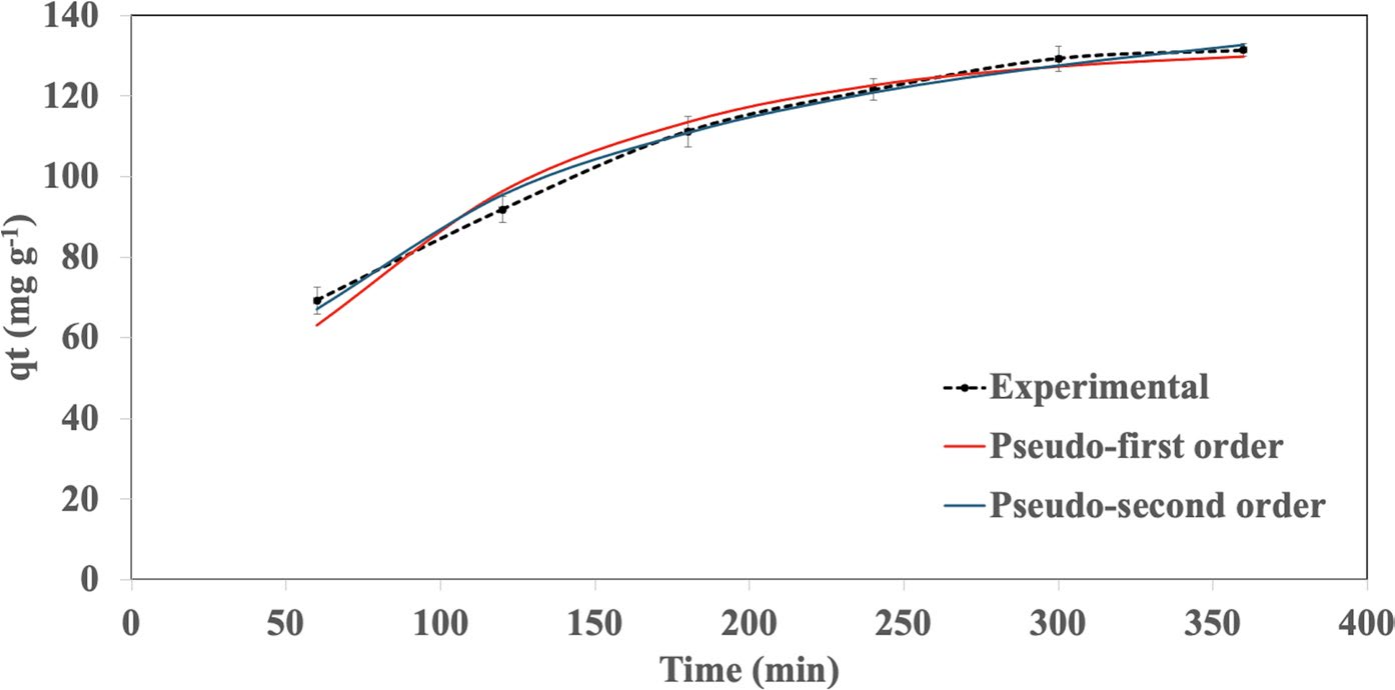
Biosorption kinetic models for alginate-EPS beads. Experimental data were fitted to the kinetic models through non-linear regression using Microsoft Excel Solver function. Error bars indicate standard deviation of means. q_t_ represents the amount of Pb adsorbed at a given time point

The biosorption mechanisms of the alginate-EPS beads are best described by the Langmuir isotherm model and the pseudo-second order kinetic model. For the isotherm models, the experimental data for Pb removal by the alginate-EPS beads showed high correlation to Langmuir isotherm model (R^2^ = 0.992) compared to Freundlich (R^2^ = 0.957) (Fig. 4, Table 1). For the kinetic models, the experimental data for Pb removal by the alginate-EPS beads showed higher correlation to pseudo-second order model (R^2^ = 0.995) compared to the pseudo-first order model (R^2^ = 0.984) (Fig. 5, Table 1). Hence, the biosorption of heavy metals by the alginate-EPS beads follows the characteristics of Langmuir isotherm and pseudo-second order kinetic.

**Table 1:** Parameters calculated from the biosorption isotherm and kinetic models for alginate-EPS beads

Model	Parameters	Values
Freundlich isotherm	n	2.758
	K_F_ (L g^−1^)	62.82
	R^2^	0.957
Langmuir isotherm	q_max_ (mg g^−1^)	556.7
	K_L_ (L mg^−1^)	0.020
	R^2^	0.992
Pseudo-first order kinetic	q_e_ (mg g^−1^)	132.6
	k_1_ (min^−1^)	0.011
	R^2^	0.984
Pseudo-second order kinetic	q_e_ (mg g^−1^)	164.8
	k_2_ (g mg^−1^ min)	6.90 × 10^–5^
	R^2^	0.995

### Packed-bed biosorption studies

#### Effect of initial pH of influent metal solution

**Fig. 6:**
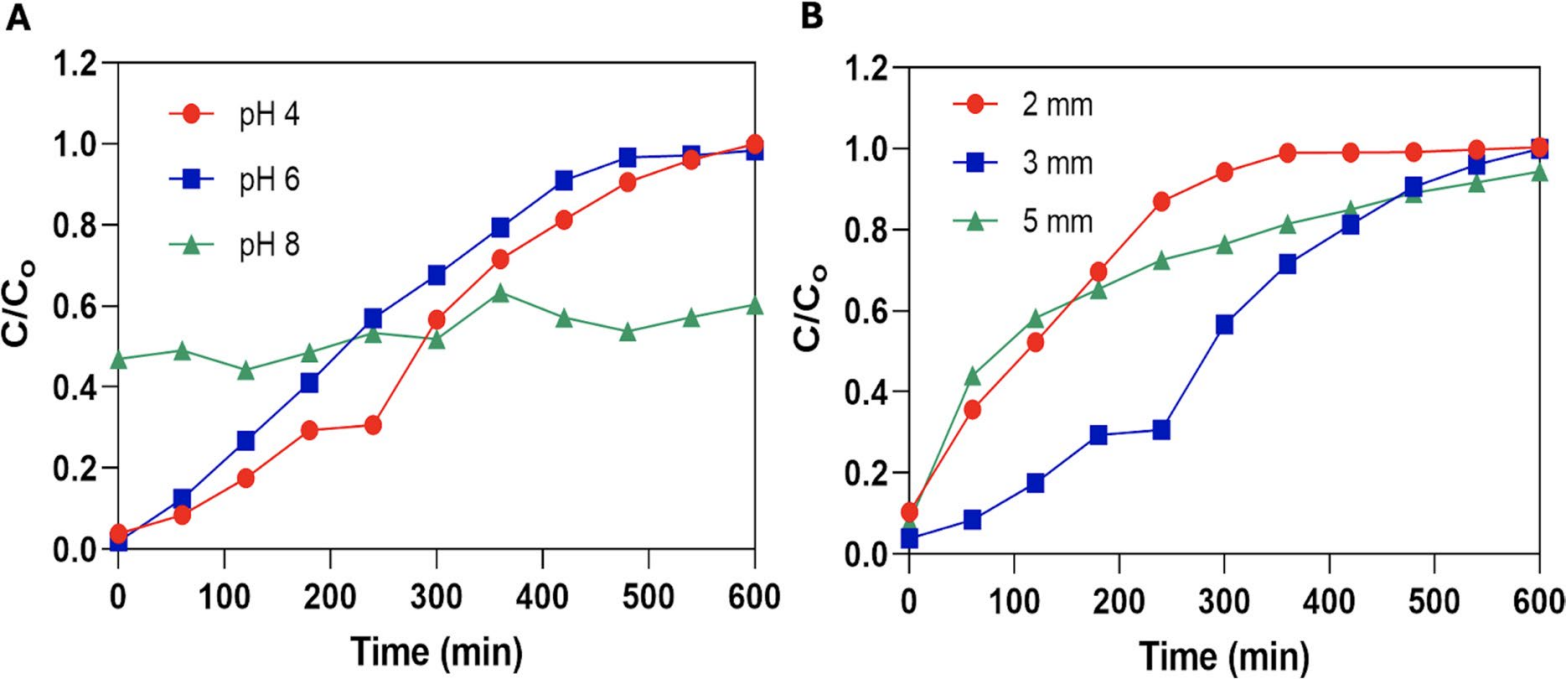
Pb biosorption breakthrough curves under the influence of **A** influent metal solutions at different initial pH values (4, 6, and 8), and **B** using alginate-EPS beads of different diameter sizes (2, 3, and 5 mm)

Results revealed that Pb removal using alginate-EPS beads was the highest for solutions with initial pH of 4 (51.52%), followed by pH 6 (48.62%) then pH 8 (46.78%) (Fig. 6A, Table 2). It is interesting to note that the breakthrough curve for metal solution with pH 8 did not reflect the typical S-shaped curve (Fig. 6A). Instead, the breakthrough curve for metal solution with pH 8 was only observed at *C/C*_*o*_ of approximately 0.4 and continued to fluctuate between 0.4 and 0.6 for the rest of the experimental period. Pb solutions with pH 8 also harboured residual precipitates, likely the result of microprecipitation of Pb under high pH condition (pH 8). By contrast, the *C/C*_*o*_ of the breakthrough curves for metal solution at pH 4 and pH 6 were detected at *C/C*_*o*_ close to 0, gradually reaching 1.0 towards the end of the experiment, which identifies with the representation of a typical breakthrough curve. When analysed together with the column exhaustion time, it was observed that the column used for solutions with initial pH 6 reached exhaustion (*C/C*_*o*_ = 0.95, *t*_*ex*_ = 470 min) sooner than for solutions with an initial pH of 4 (*t*_*ex*_ = 540 min) (Fig. 6A). This suggested that the alginate-EPS beads packed-bed column was able to continuously perform biosorption of Pb ions for a longer duration when the metal solution has an initial pH of 4 compared to pH 6. On the contrary, the alginate-EPS beads packed-bed column with metal solution at pH 8 did not reach exhaustion within the 600 min duration. This suggested that the column may not have adsorbed the Pb ions efficiently, and lesser surface areas were occupied by Pb ions. This may also be linked to the mass transfer limitation seen from the microprecipitation observed in influents with pH 8. Overall, column biosorption was optimum when the initial pH of the metal solution was at pH 4 with longer exhaustion time (*t*_*ex*_ = 540 min) and higher percentage of Pb removed (51.52%).

**Table 2:** Parameters of breakthrough curves from packed-bed biosorption with Pb solutions with different initial pH

pH	*t*_*ex*_ (min)	Pb uptake, *q*_*e*_ ( mg g^−1^)	Pb removal (%)
4	540	13.29 ± 0.08^a^	51.52 ± 0.30^a^
6	470	10.91 ± 0.01^b^	48.62 ± 0.02^b^
8	-	13.40 ± 0.01^a^	46.78 ± 0.04^c^

Mean values with standard deviations are presented for Pb uptake (*q*_*e*_) and Pb removal. Means with the same letter within a column indicate no significant difference according to Tukey’s Test (*p* < 0.01)

#### Effect of biosorbent size

Results revealed that Pb removal was the highest when using 3 mm-sized beads (51.52%), followed by 2 mm-sized beads (39.23%) and finally, with 5 mm-sized beads (28.52%) (Fig. 6B, Table 3). The highest Pb uptake was also achieved by the use of 3 mm-sized beads (13.29 mg g^−1^), but was followed by 5 mm-sized beads (8.17 mg g^−1^), with 2 mm-sized beads having the lowest Pb uptake (5.99 mg g^−1^). The bead size influenced the exhaustion time as well, with columns packed with 5 mm-sized beads as the slowest to reach exhaustion (*t*_*ex*_ = 600 min), followed by 3 mm-sized beads (*t*_*ex*_ = 540 min) then 2 mm-sized beads (*t*_*ex*_ = 320 min). In short, column biosorption was most optimum with 3 mm-sized beads as the highest Pb removal (51.52%) and Pb uptake (13.29 mg g^−1^) was recorded (Fig. 6B). For the 5 mm-sized beads, despite having the longest column exhaustion time (*t*_*ex*_*),* the percentage of Pb removed was the lowest. As such, the ideal bead size for Pb biosorption was identified as 3 mm.

**Table 3:** Parameters of breakthrough curves from packed-bed biosorption with different diameter size of alginate-EPS beads

Bead diameter (mm)	*t*_*ex*_ (min)	Pb uptake, *q*_*e*_ (mg g^−1^)	Pb removal (%)
2	320	5.99 ± 0.00^c^	39.23 ± 0.02^b^
3	540	13.29 ± 0.08^a^	51.52 ± 0.30^a^
5	600	8.17 ± 0.00^b^	28.52 ± 0.01^c^

Mean values with standard deviations are presented for Pb uptake (*q*_*e*_) and Pb removal. Means with the same letter within a column indicate no significant difference according to Tukey’s Test (*p* < 0.01)

#### Column regeneration

**Fig. 7:**
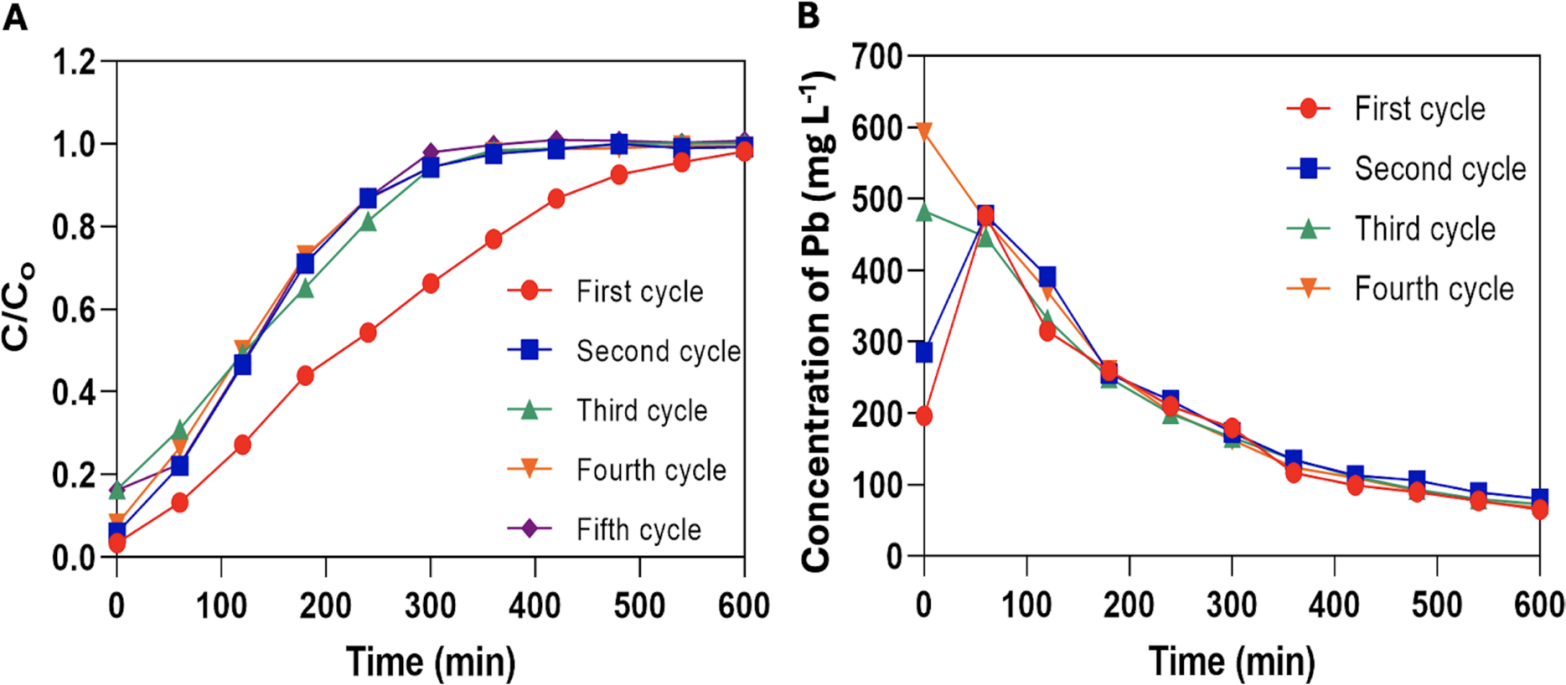
**A** Pb biosorption breakthrough curves for five adsorption cycles and the **B** Pb recovery curves from four desorption cycles

Breakthrough curves from the adsorption cycles (Fig. 7A) revealed the ability of alginate-EPS beads to be regenerated up to five times. It is interesting to note that the breakthrough curves from the re-adsorption cycles (second to fifth cycles) have similar curves to the first adsorption cycle. However, all four re-adsorption cycles reached column exhaustion (*C/C*_*o*_ = 0.95) sooner than the first adsorption cycle. The Pb concentrations from the desorption cycles (Fig. 7B) revealed that most of the Pb ions were eluted at the beginning of the desorption as the highest Pb levels were detected in the effluent before 100 min. The Pb levels then gradually decrease to as low as 100 mg L^−1^ at the end of the desorption experiment (t = 600 min) for all four desorption cycles.

The percentage of Pb removed (Pb removal) from all five adsorption cycles remained fairly consistent (from 41 to 51%) (Table 4). However, Pb uptake by the EPS beads was highest in the first adsorption cycle (13.29 mg g^−1^) and decreases gradually over the consecutive cycles (6.62, 6.40, 6.33, and 6.15 mg g^−1^). On the contrary, the percentage of Pb recovered from desorption (Pb recovery) was highest in the fourth cycle (132.46%), followed by the third cycle (124.57%), the second cycle (123.37%), and the first cycle (66.47%). This suggested that higher Pb recoveries were found in the subsequent desorption cycles compared to the first desorption cycle. Therefore, it was concluded that the alginate-EPS beads can be successfully regenerated for up to five adsorption cycles with consistent Pb removal and high recovery of the biosorbed Pb ions.

**Table 4:** Parameters of breakthrough curves from packed-bed adsorption–desorption cycles

Adsorption cycle	*t*_*ex*_ (min)	Pb uptake, *q*_*e*_ (mg g^−1^)	Pb removal (%)	Pb recovery (%)
First	550	13.29 ± 0.08^a^	50.59 ± 0.29^a^	66.47 ± 0.11^d^
Second	320	6.62 ± 0.01^b^	43.33 ± 0.05^c^	123.37 ± 0.01^c^
Third	320	6.40 ± 0.01^c^	41.89 ± 0.05^d^	124.57 ± 0.08^b^
Fourth	320	6.33 ± 0.01^c^	41.40 ± 0.04^e^	132.46 ± 0.12^a^
Fifth	290	6.15 ± 0.00^d^	44.43 ± 0.02^b^	-

Mean values with standard deviations are presented for Pb uptake (*q*_*e*_), Pb removal, and Pb recovery. Means with the same letter within a column indicate no significant difference according to Tukey’s Test (*p* < 0.01)

**Fig. 8:**
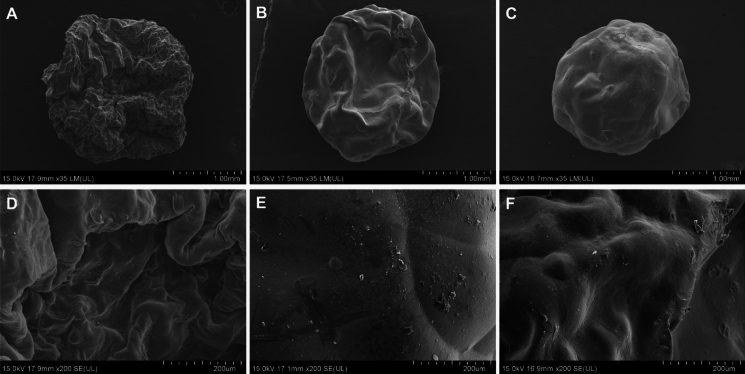
SEM micrographs of alginate-EPS beads at 35 × magnification: **A** before desorption, **B** after first cycle of desorption, and **C** after fourth cycle of desorption. SEM micrographs of alginate-EPS beads at 200 × magnification: **D** before desorption, **E** after first cycle of desorption, and **F** after fourth cycle of desorption

The surface morphology of the alginate-EPS beads revealed rough and uneven folds on the surface, and this was observed for beads prior to any adsorption or desorption cycles (Fig. 8A, D). After the first desorption cycle, the surface of the beads became smoother, and there were lesser uneven folds found on the surface (Fig. 8B and E). After the fourth desorption cycle, the surface of the beads have smoothen rather evenly (Fig. 8C and F) compared to beads prior to desorption and from earlier cycles of desorption. Nevertheless, perforations can be observed on the surface of the beads, most likely caused by the acidic desorption agent (1% CaCl_2_, pH 3). This was detected on the surface of beads after the first and fourth desorption with pore sizes ranging from 20 to 40 µm (200 × magnification) (Fig. 8E and F). These morphological changes (i.e., smoother surfaces, perforations) after desorption may have contributed to the lower Pb uptake reported from the second to fifth re-adsorption cycles, compared to the first adsorption cycle. This is because metal biosorption is very much dependent on sorption of metal cations to the surface of the biosorbent, hence, changes to the surface may have significant influence on amount of metal cations adsorbed.

## Discussion

### Characterization of alginate-EPS beads

The surface morphology of the alginate-EPS beads was observed to have uneven folds, likely the result of the reaction between sodium alginate and calcium chloride to form calcium alginate. This creates a gel outer layer with a porous structure due to the crosslinking network of calcium alginate polymer chains with each other (Ayarza et al. 2017). The encapsulation of EPS in the alginate beads presents a heterogonous distribution of EPS throughout the beads, contributing further to the formation of uneven surfaces and folds (Dobrowolski et al. 2019; Cheah et al. 2022b). As this study did not anticipate EPS to alter surface area, direct measurements of the surface area of the alginate-EPS beads was not performed. However, it was postulated that the folds on the surface observed may likely contribute to greater surface area. These rough and uneven structures on the alginate-EPS beads may likely benefit biosorption of heavy metals such as Pb, as they increase the total surface area of the beads (Nasrullah et al. 2018). This leads to more exposed metal binding sites, thereby increasing the biosorption capacity of the alginate-EPS beads.

The functional groups detected from the alginate-EPS beads were consistent with other studies (Xu et al. 2021; Cheah et al. 2022b). The presence of these various functional groups facilitates Pb biosorption through the binding of Pb cations to hydroxyl (–OH), carboxyl (COOH) groups, and in some cases ascribed to the possible presence of phosphate (PO_4_^3−^), which are known to bind strongly to metal cations (Southichak et al. 2006; Nadeem et al. 2008; Cerino-Córdova et al. 2013; Cheah et al. 2022b). The aforementioned groups likely originated from compounds found in alginate or bacterial EPS from the alginate-EPS beads. These functional groups support binding of metal cations such as Pb as they provide a negatively charged surface on the beads, which can then electrostatically attract and adsorb the positively charged metal cations (Gupta and Diwan 2017; Cheah et al. 2022b). Therefore, the abundance of these functional groups detected on the EPS beads may have contributed to their efficacy in biosorbing Pb.

### Biosorption of Pb by plain alginate and alginate-EPS beads

This study revealed that EPS integrated into alginate beads were able to enhance Pb removal for the biosorbents. This observation agreed with previous studies conducted using alginate-EPS beads, whereby the role of EPS in enhancing metal removal was observed for most metal cations (Cu, Pb, Zn, Cd) (Cheah et al. 2022a, 2022b). In the earlier reports, the efficiency of metal biosorption was ascribed to the presence of various functional groups present on alginate-EPS beads. As such, the role of functional groups is very likely replicated for the alginate-EPS beads observed in this study. The superiority of alginate-EPS beads compared to plain alginate beads in sorption capacity and in lowering environmental footprint can perhaps be investigated in future studies, as this requires comparisons of alginate-EPS beads in optimized and ready-to-use forms.

### Biosorption mechanisms

The isotherm studies indicated that the biosorption mechanism of the alginate-EPS beads complied with the characteristics described by the Langmuir isotherm. As such, it is concluded that the biosorption of Pb using alginate-EPS beads existed as a monolayer adsorption, where each adsorbed Pb ions were in direct contact with the binding sites, providing a strong electrostatic attraction (Chen 2015; Liu et al. 2019). Compliance to Langmuir model also conveyed that the alginate-EPS beads have a finite number of sorption sites on the alginate-EPS beads, and these sorption sites can become saturated with adsorbed metal ions (Kumari eta l. 2017; Li et al. 2022). With the sorption sites becoming saturated, the beads will no longer be able to adsorb further any metal ions, thus limiting the uptake and sorption of Pb. This may also influence the exhaustion time and regeneration of the alginate-EPS beads when used as packed-bed columns in the subsequent studies.

In addition, the biosorption mechanism of the alginate-EPS beads also complied with the characteristics described by the pseudo-second order kinetic model. This suggested that the biosorption of Pb ions is limited by chemisorption, aligned to several earlier studies using alginate-EPS beads (Cheah et al. 2022a, 2022b). The rate limiting step occurs when active binding sites are saturated by the Pb cations. As such, it is concluded that the biosorption of Pb ions on the alginate-EPS beads occurred through a strong chemical bond (Wei et al. 2016). This also supports the monolayer adsorption described by the Langmuir isotherm, whereby compliance to Langmuir indicates that Pb ions are bound to the metal binding sites through a strong chemical interaction. This further validated that alginate-EPS beads are strong biosorbents to sequester heavy metals from wastewater.

### Packed-bed biosorption studies

#### Effect of initial pH of influent metal solution

The biosorption of Pb was clearly affected by the initial pH of the influent metal solution. The removal of Pb was the highest for metal solutions with initial pH of 4 compared to pH 6 and pH 8. It has been reported that at pH 4, the acidic condition allows the Pb metals in the solution to remain in their ionic forms (Sheoran and Sheoran 2006), and these ionic forms can be readily adsorbed by the alginate-EPS beads. The ionic forms of Pb enables efficient electrostatic binding of positively charged Pb ions to the negatively charged functional groups on the beads (Mehta and Gaur 2005). This, however, is more challenging with increasing pH values, as metals are known to precipitate in alkaline conditions due to the formation of insoluble metal-hydroxide complexes (Chen et al. 2018; Cheah et al. 2022b).

Therefore, it is not surprising that at pH 6, a lower percentage of Pb is removed as some Pb ions would have undergone initial stages of precipitation, thereby decreasing the amount of readily dissolved Pb ions that can be adsorbed by the alginate-EPS beads. This is further evidenced by Pb removal from metal solutions of pH 8, which was the lowest compared to pH 4 and pH 6. Additionally, the breakthrough curve plotted from metal solution with pH 8 was different compared to the breakthrough curves for biosorption of Pb for solutions with pH 4 and pH 6. In higher pH conditions, it is likely that a large portion of the Pb ions has precipitated, observed typically as microprecipitation in the influent solution. With Pb ions forming precipitates, there are less Pb ions in the influent solution to be passed through the column, decreasing adsorption. The Pb levels from the column effluent must be taken into consideration for realistic application. The concentration of heavy metals at the column outlet needs to be low enough to be considered safe before disposal. Therefore, it is ideal to have near to complete removal of metal when the treated solution first elutes the column, which is why the S-shaped breakthrough curve is often preferred (Patel 2019). Considering this, the biosorption of Pb by the alginate-EPS beads packed-bed column is most efficiently achieved when the metal solution (influent) is at pH 4.

#### Effect of biosorbent size

The removal of Pb in the packed-bed system was clearly affected by the size of alginate-EPS beads as the percentages of Pb removed were significantly different between the different sizes of beads used in this study. However, the results did not indicate any specific trends between biosorbent size and Pb removal as the Pb removal did not increase or decrease accordingly when larger-sized alginate-EPS beads were used. The findings here, using packed-bed column, differed from other batch system studies. In batch systems, smaller-sized biosorbents compared to larger-sized biosorbents will generally yield a higher biosorption efficiency due to larger total surface area for adsorption (Gulnaz et al. 2005; Mishra et al. 2010; Kelly-Vargas et al. 2012). On the contrary, packed-bed systems did not show preference to specific sizes of the alginate-EPS beads used, although it is postulated that the size of biosorbents may affect the packing density of the column (Unger et al. 2008; Patel 2019). Therefore, the smaller-sized biosorbents would be packed more densely in the column, while larger-sized biosorbents would be packed more loosely. A column that is packed more densely has less void volume in between the biosorbent particles, which decreases the surface exposure of the biosorbents leading to lower diffusion of metal ions (Kumar et al. 2016). Therefore, despite the higher total surface area generated from using smaller sized beads, when packed densely, not all surfaces from the beads will be exposed for Pb adsorption. In addition, a densely packed column may introduce fluid resistance when the metal solution flows through the column as there is less void volume. This creates high pressure within the column which may force the metal solution to pass quicker through the column (Hatzikioseyian et al. 2001), thereby decreasing the interaction time of the Pb ions with the beads. The reduced surface exposure from the beads and a lower interaction time may have possibly led to the lower Pb removal efficiency by 2 mm-sized beads compared to the 3 mm-sized beads.

The column with 5 mm-sized beads was the slowest to reach exhaustion time. This is consistent with reports that when metal diffusivity to biosorbent is reduced, the column exhaustion time is increased (Mehta and Gaur 2005; Kumar et al. 2016). With larger sized beads, the total surface area is reduced, leading to a slower diffusion rate of Pb ions to the binding sites. This led to the reduced overall percentage of Pb removal by the column. Hence, considering the Pb removal efficacy, the 3 mm-sized alginate-EPS beads were established as the most suitable bead size recommended for column packing for Pb removal.

#### Column regeneration

Results from the five adsorption cycles in this study indicated that the alginate-EPS beads are capable for multiple regeneration. Despite a decrease in Pb uptake (*q*_*e*_) after the first adsorption cycle, the overall percentage of Pb removed remained consistent for all the cycles. Similar results were also reported by Ajao et al. (2020) and Ronda et al. (2015), where the adsorption of metal and column exhaustion time decreased after the first desorption cycle. This is likely due to the acid treatment from the desorption agent, which may have changed the structure or characteristics of the biosorbent (Sulaymon et al. 2014). This was evident in the SEM analysis of the alginate-EPS beads sampled from before and after desorption cycles using acidified CaCl_2_. After desorption, the surface of the beads appeared smoother, which may have led to a lower total surface area for metal adsorption and consequently a reduced overall Pb uptake. Additionally the presence of perforations on the bead surfaces is an indication of surface damage from chemical treatment (Cheah et al. 2020). This may have implicated the metal binding sites on the surface of the beads which are critical for adsorption of Pb ions (Mehta and Gaur 2005), reducing Pb uptake in the re-adsorption cycles. Nevertheless, the overall percentage of Pb removed was not severely compromised by desorption using acidified CaCl_2_, as all five adsorption cycles were able to remove between 41.40 and 50.59% of Pb from the solution. It is also possible that Pb accumulation may occur over time, possibly beyond the five cycles of usage tested in this study, as efficiency of adsorbing-desorbing Pb from the column can be compromised by the repeated cycles of exposure to weak acids.

It was also noted that the first desorption cycle was not able to recover the adsorbed Pb completely, as the recovery was less than 100%. This suggested that some of the binding sites on the EPS beads remained bound to Pb ions prior to the second adsorption cycle. The second, third, and fourth desorption cycles showed Pb recoveries of more than 100%, indicating that the Pb ions retained in the column from previous cycles were eluted in subsequent desorption cycles. This further suggested that the acidified CaCl_2_ was not able to completely desorb the retained Pb ions in the first desorption cycle. Nevertheless, desorption can also be optimized for maximum recovery of metals by adjusting the relevant column parameters. One such parameter is the flow rate of the desorption agent (Volesky et al. 2003), a parameter that is still unexplored in current literatures. Future optimization studies are suggested using the same desorption agent (acidified CaCl_2_) but under different flowrates to achieve complete recovery of the retained metals and in turn, maximizing the re-adsorption of metals in the following cycle. The amenability of alginate-EPS beads packed bed column to sorption–desorption process highlights the regeneration potential of the column for reuse (Pandey et al. 2007). This establishes the alginate-EPS beads as sustainable biosorbents as they are reusable after regeneration.

The findings from this study may be specific to the experimental conditions tested, therefore the feasibility of the alginate-EPS beads in real world settings remains to be explored. It is possible that while this study showed that ideal pH conditions were pH 4, the natural wastewaters may be of varying pH conditions that affect biosorption. The presence of other pollutants such as metals and organic matter may also implicate sorption efficiency. As such, the ability to replicate the efficacy of alginate-EPS beads remains to be investigated. Likewise, the regeneration ability of the alginate beads may also differ according to the profile of the natural wastewaters. Further studies such as the optimization of alginate-EPS beads are therefore critical for gradual applications.

## Conclusion

Alginate-EPS beads were revealed to be effective biosorbents for removal of heavy metals in this study. Characterization studies of the alginate-EPS beads revealed uneven surface morphology and an abundance of organic functional groups which suggest a promising biosorption capacity. The mechanism of biosorption of the alginate-EPS beads suggested a strong chemical interaction between the metal ions and metal binding sites, which supported the hybrid beads as suitable biosorbents for heavy metal removal. When applied in a packed-bed system for biosorption of Pb, effects of important process parameters (pH of influent metal solution, biosorbent size) were revealed, and the parameters were optimized for maximum Pb removal. Column regeneration was successfully performed up to five adsorption cycles using acidified CaCl_2_ as the desorption agent with consistent Pb removal in all cycles. This study highlighted the potential of alginate-EPS beads for removal of heavy metals, and elucidated the often-overlooked but important parameters (i.e., initial pH of influent, bead size) which influenced the biosorption process by packed-bed columns. Alginate-EPS beads have also shown good potential for regeneration and recovery of adsorbed metals, hence they are useful biosorbents in packed-bed systems that can be established as a feasible and sustainable alternative to remove heavy metals in wastewater treatment.

## Data Availability

The data will be shared on request to the corresponding author.
